# Evaluation of gene expression cassettes and production of poly(3-hydroxybutyrate-co-3-hydroxyhexanoate) with a fine modulated monomer composition by using it in *Cupriavidus necator*

**DOI:** 10.1186/s12934-016-0583-7

**Published:** 2016-10-28

**Authors:** Hisashi Arikawa, Keiji Matsumoto

**Affiliations:** GP Group, Corporate R&D Planning and Administration Division, KANEKA CORPORATION, 1-8 Miyamae-Cho, Takasago-Cho, Takasago, Hyogo 676-8688 Japan

**Keywords:** Polyhydroxyalkanoates, PHBHHx, *Cupriavidus necator*, Promoter, Ribosome binding site

## Abstract

**Background:**

*Cupriavidus necator* has attracted much attention as a platform for the production of polyhydroxyalkanoate (PHA) and other useful materials. Therefore, an appropriate modulation of gene expression is needed for producing the desired materials effectively. However, there is insufficient information on the genetic engineering techniques required for this in *C. necator*.

**Results:**

We found that the disruption of a potential ribosome binding site (RBS) in the *phaC1* gene in *C. necator* caused a small decrease in the PhaC1 expression level. We applied this result to finely regulate the expression of other genes. Several gene expression cassettes were constructed by combining three *Escherichia coli* derived promoters (P_lacUV5_, P_trc_ and P_trp_) to the potential RBS of *phaC1* or its disruptant, respectively. Their expression levels were then determined via a *lacZ* reporter assay in *C. necator* strains. The promoter strengths were both ranked similarly for the cells that were cultured with fructose or palm kernel oil as a sole carbon source (P_trc_ ≥ P_lacUV5_ > P_trp_), both of which were much stronger than the *phaC1* promoter. The disruption of RBS had minute attenuation effect on the expression level of these expression cassettes with *E. coli* promoters. Furthermore, they were used to finely regulate the (*R*)-3-hydroxyhexanoate (3HHx) monomer ratio in the production of poly[(*R*)-3-hydroxybutyrate-*co*-3-hydroxyhexanoate] (PHBHHx) via *R*-specific enoyl-CoA hydratases (PhaJs). The 3HHx composition in PHBHHx is crucial because it defines the thermal and mechanical properties of the resulting plastic material. The *C. necator* mutant strains, whose PhaJ expression was controlled under the gene expression cassettes, could be used to produce PHBHHx with various 3HHx compositions in the same culture conditions.

**Conclusions:**

We constructed and evaluated several gene expression cassettes consisting of promoters and RBSs that finely regulate transcription and translation. These were then applied to finely modulate the monomer composition in the production of PHBHHx by recombinant *C. necator*.

**Electronic supplementary material:**

The online version of this article (doi:10.1186/s12934-016-0583-7) contains supplementary material, which is available to authorized users.

## Background

*Cupriavidus necator* (formerly *Ralstonia eutropha*) is a non-pathogenic gram-negative soil bacterium of the β-proteobacteria class. It is a well-known model organism for studying polyhydroxyalkanoate (PHA) synthesis and accumulation [1]. PHA has attracted industrial attention as an environmentally friendly material because it exhibits complete biodegradability and is a possible alternative to petroleum-based plastics [2]. Poly[(*R*)-3-hydroxybutyrate] (PHB) is the most widely investigated member of the PHA family. *Cupriavidus necator* can greatly accumulate PHB, which can constitute up to 80% of its dry cell weight, from renewable carbon sources [3]. Furthermore, the recombinant strains harboring the PHA synthase gene derived from *Aeromonas caviae* FA440 can produce poly[(*R*)-3-hydroxybutyrate-*co*-3-hydroxyhexanoate] (PHBHHx) with improved physical properties [4]. PHBHHx is more flexible than PHB, and its flexibility can be regulated by its monomer composition [5]. Increasing incorporation of (*R*)-3-hydroxyhexanoate (3HHx) as a second monomer results in increased flexibility of the copolymer. This characteristic is expected to be important for the practical application in many fields.

There has been a recent focus on *C. necator* as a host strain for the production of proteins and other metabolites. Srinivasan et al. reported that high yields of organophosphohydrolase protein could be obtained without the formation of inclusion bodies due to high levels of gene expression in high-cell density fermentation [6, 7]. In addition, some studies demonstrated that other useful materials such as cyanophycin, branched-chain alcohols, methyl ketones, or medium-chain-length fatty acids could be produced by genetically engineering *C. necato*r strains to express various exogenous genes [8–11].

To improve the genetic construction of biosynthetic pathways, various expression systems in *C. necator* based on promoters and plasmid vectors have been evaluated. For instance, some popular and native promoters such as P_lac_, P_lacUV5_, P_tac_, T7, P_BAD_, P_phaC1_, P_phaP_, P_acoE_, P_acoD_, P_acoX_, and P_pdhE_, were investigated. Furthermore, P_tac_, which is known to be a constitutive strong promoter in *Escherichia coli*, functions well also in *C. necator* [12–16]. Additionally, highly stable vectors even under no antibiotic pressure have also been developed since various plasmid vectors are very unstable in *C. necator* H16. Sato et al. showed that artificial plasmids containing the partition locus and oriV28 region derived from the *Cupriavidus metallidurans* CH34 megaplasmid are highly stable in *C. necator* [17]. Moreover, Gruber et al. reported that the RP4 partitioning system confers stability on plasmids vectors [18]. They developed new plasmid vectors with varying combinations of replication origins and promoters, and demonstrated that the minireplicon derived from RSF1010 and the bacteriophage T5 promoter are superior in terms of stability and the expression level. It is also worthy to note that the T5 promoter is much stronger than P_tac_.

In evaluating expression systems, greater significance is generally placed on the systems yielding higher intensity expression levels. However, if the expression is too strong, cell growth or the solubility of the resulting protein may be adversely affected. In addition, appropriately modulating gene expression is also required for fermentative production of materials that can be effectively produced through fine control of flux of their metabolic intermediates. Therefore, in the present study, we developed a gene expression cassette by combining common promoters derived from *E. coli*, P_lacUV5_, P_trc_, and P_trp_, in conjunction with a potential ribosome binding site (RBS) derived from the region upstream of *phaC1*_*Cn*_ or its disruptant. The expression levels of each cassette were examined via a reporter gene assay in *C. necator* recombinant strains and compared to P_phaC1_ combined with its native potential RBS or disruptant. Furthermore, these promoter-RBS gene expression cassettes were used to regulate the monomer ratio of PHBHHx produced in the recombinant *C. necator*.

## Results

### Evaluation of promoter-RBS gene expression cassettes in *C. necator*

**Fig. 1:**
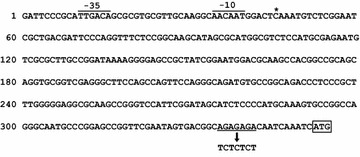
Nucleotide sequence of the 5′ upstream region of the *phaC1* gene (P_phaC1_RBS). The promoter location, transcriptional start site, and translation initiation codon have been experimentally identified previously [19]. The putative −35 and −10 promoter region are overlined and labeled. An *asterisk* indicates the transcriptional start site. The translation initiation codon is *boxed*. The potential ribosome binding site (RBS) is *underlined*, and the *arrow* indicates the disrupted RBS sequence in P_phaC1_dRBS

The sequence AGAGAGA located 11 bases upstream of the *C. necator* PHA synthase (*phaC1*) gene initiation codon has been shown as a potential RBS [19]. To ascertain the function of this sequence, an expression plasmid with PhaC1 under the control of its native promoter in combination with its potential RBS (P_phaC1_RBS) or RBS disruptant (P_phaC1_dRBS) were constructed (Fig. 1) and introduced into the recombinant *C. necator* strain, H16/ds. Table 1 summarizes the bacterial strains and plasmids used in this study. The H16/ds strain is a *phaC1* disruptant of the *C. necator* H16 strain, which was previously shown to be unable to produce PHAs [17].

**Table 1:** Bacterial strains and gene expression plasmids

Strain or plasmid	Description^a^	Source or reference
*C. necator*		
H16	Wild type	ATCC17699
H16/ds	H16 derivative; Δ*phaC1*	17
005dZ126	H16 derivative; Δ*phaC1*::*phaC*_*Ac*_NSDG, Δ*phaZ1*, Δ*phaZ2*, Δ*phaZ6*	27
CnC1A	005dZ126, P_phaC1_RBS-*phaJ4a*	This study
CnTRPDA	005dZ126, P_trp_dRBS-*phaJ4a*	This study
CnTRPA	005dZ126, P_trp_RBS-*phaJ4a*	This study
CnUV5DA	005dZ126, P_lacUV5_dRBS-*phaJ4a*	This study
CnUV5A	005dZ126, P_lacUV5_RBS-*phaJ4a*	This study
CnTRCDA	005dZ126, P_trc_dRBS-*phaJ4a*	This study
CnC1DB	005dZ126, P_phaC1_dRBS-*phaJ4b*	This study
CnC1B	005dZ126, P_phaC1_RBS-*phaJ4b*	This study
CnTRPB	005dZ126, P_trp_RBS-*phaJ4b*	This study
CnUV5B	005dZ126, P_lacUV5_RBS-*phaJ4b*	This study
CnTRCB	005dZ126, P_trc_RBS-*phaJ4b*	This study
*E. coli*		
JM109	*recA1 endA1 gyrA96 thi hsdR17 supE44 relA1* Δ(*lac*-*proAB*)/F’ [*traD36 proAB* ^+^ *lacI* ^q^ *lacZ*ΔM15]	Takara
S17-1	*recA pro hsdR* RP4-2-Tc::Mu-Km::Tn7	ATCC47055
Plasmids		
pCUP3	Stable plasmid vector in *C. necator*, Km^r^	17
pCUP3-P_phaC1_RBS-phaC1	P_phaC1_RBS-*phaC1* expression cassette cloned into pCUP3	This study
pCUP3-P_phaC1_dRBS-phaC1	P_phaC1_dRBS-*phaC1* expression cassette cloned into pCUP3	This study
pCUP3-RBS-lacZ	RBS-*lacZ* cloned into pCUP3	This study
pCUP3-P_phaC1_RBS-lacZ	P_phaC1_RBS-*lacZ* expression cassette cloned into pCUP3	This study
pCUP3-P_lacUV5_RBS-lacZ	P_lacUV5_RBS-*lacZ* expression cassette cloned into pCUP3	This study
pCUP3-P_trp_RBS-lacZ	P_trp_RBS-*lacZ* expression cassette cloned into pCUP3	This study
pCUP3-P_trc_RBS-lacZ	P_trc_RBS-*lacZ* expression cassette cloned into pCUP3	This study
pCUP3-dRBS-lacZ	dRBS-*lacZ* cloned into pCUP3	This study
pCUP3-P_phaC1_dRBS-lacZ	P_phaC1_dRBS-*lacZ* expression cassette cloned into pCUP3	This study
pCUP3-P_lacUV5_dRBS-lacZ	P_lacUV5_dRBS-*lacZ* expression cassette cloned into pCUP3	This study
pCUP3-P_trp_dRBS-lacZ	P_trp_dRBS-*lacZ* expression cassette cloned into pCUP3	This study
pCUP3-P_trc_dRBS-lacZ	P_trc_dRBS-*lacZ* expression cassette cloned into pCUP3	This study

^a^RBS, potential ribosome binding site of *phaC1*; dRBS, RBS disruptant

**Fig. 2:**
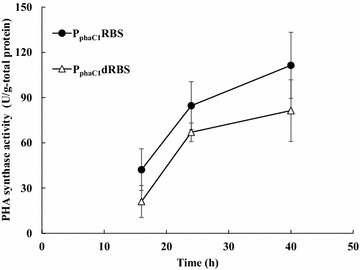
Polyhydroxyalkanoate (PHA) synthase activity. Cells were cultured in mineral salt medium with 0.129 w/v% (NH_4_)_2_SO_4_ and 1.5 w/v% palm kernel oil. The values shown are averages of triplicate experiments. *Error bars* indicate standard deviations

The strains harboring the PhaC1 expression plasmids were cultured with palm kernel oil as a carbon source, and their PHA synthase activities were measured (Fig. 2). Although P_phaC1_dRBS showed a reduction in PHA synthase activity, conveniently, it was still considered highly active since it was more than 50% of the P_phaC1_RBS activity. Therefore, we tried to apply the disruption of this potential RBS for the modulation of the gene expression.

**Fig. 3:**
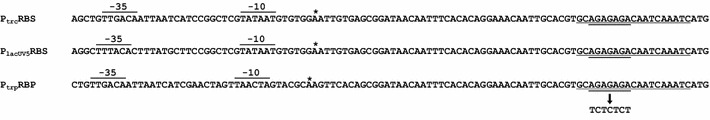
Structure of gene expression cassettes with the *E. coli* promoters, P_trc_RBS, P_lacUV5_RBS, and P_trp_RBS. The −35 and −10 promoter regions are overlined and labeled. *Asterisks* indicate the transcriptional start sites in *E. coli*. The 19-bp sequences derived from the region upstream of *phaC1* are *underlined*, and the potential ribosome binding sites (RBS) are *double-underlined*. In their RBS disruptants, P_trc_dRBS, P_lacUV5_dRBS, and P_trp_dRBS, the potential RBS sequence (AGAGAGA) was changed to TCTCTCT

For the construction of the promoter-RBS gene expression cassettes, a 19-bp sequence (GCAGAGAGACAATCAAATC) containing the potential RBS derived from the region upstream of the *phaC1* gene, or its RBS disruptant, were attached to the *E. coli* promoters (Fig. 3). Then, the plasmids with LacZ under the control of the resulting cassettes were constructed and introduced into the 005dZ126 strain, which is the *C. necator* H16 derivative native PHA synthase gene substituted with PHBHHx synthase gene.

**Fig. 4:**
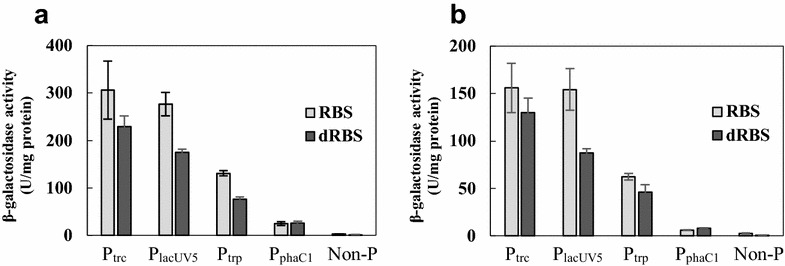
Activity of gene expression cassettes determined by *lacZ* reporter assay. Cells were grown with fructose (**a**) or palm kernel oil (**b**) as a sole carbon source. Triplicate samples from each of the two independent experiments were measured by optical absorbance (at 570 nm) of the chromogenic product, chlorophenol red. Data are presented as mean with *error bars* indicating the standard deviation. Non-P, non-promoter construct; RBS, with potential ribosome binding site of *phaC1*; dRBS, RBS disruptant

First, the recombinant strains harboring the *lacZ* gene cloned downstream of the expression cassette with the potential RBS (AGAGAGA) were cultured with fructose or palm kernel oil as a sole carbon source, and the promoter strengths were compared with that of P_phaC1_ by measuring the resulting β-galactosidase activity (Fig. 4). Although there was about a twofold higher activity with fructose than with palm kernel oil, the rank order of the promoter strengths in *C. necator* was the same (P_trc_ ≥ P_lacUV5_ > P_trp_ > P_phaC1_) in both conditions. The strongest promoter, P_trc_, exhibited expression that was at least 20-fold higher than that of P_phaC1_ with palm kernel oil. Finally, as expected, promoter-less constructs had minimal activity.

Secondly, the effects of RBS disruption on gene expression were also examined (Fig. 4). The RBS-disrupted expression cassettes with the *E. coli* promoters, and even non-promoter construct, showed a decline in the activity. The diminution rate was not significant as with the PHA synthase activity in P_phaC1_dRBS (Fig. 2), and the RBS disruptants with the *E. coli* promoters still maintained the activity of more than 55%, respectively. On the other hand, by disrupting the potential RBS with P_phaC1_, the β-galactosidase activity was not changed or slightly increased in both culture conditions unexpectedly. Since attenuation was not observed, this suggests that the potential RBS sequence did not function as a RBS in this construct.

### Controlling 3HHx composition of PHBHHx using the expression cassettes

**Fig. 5:**
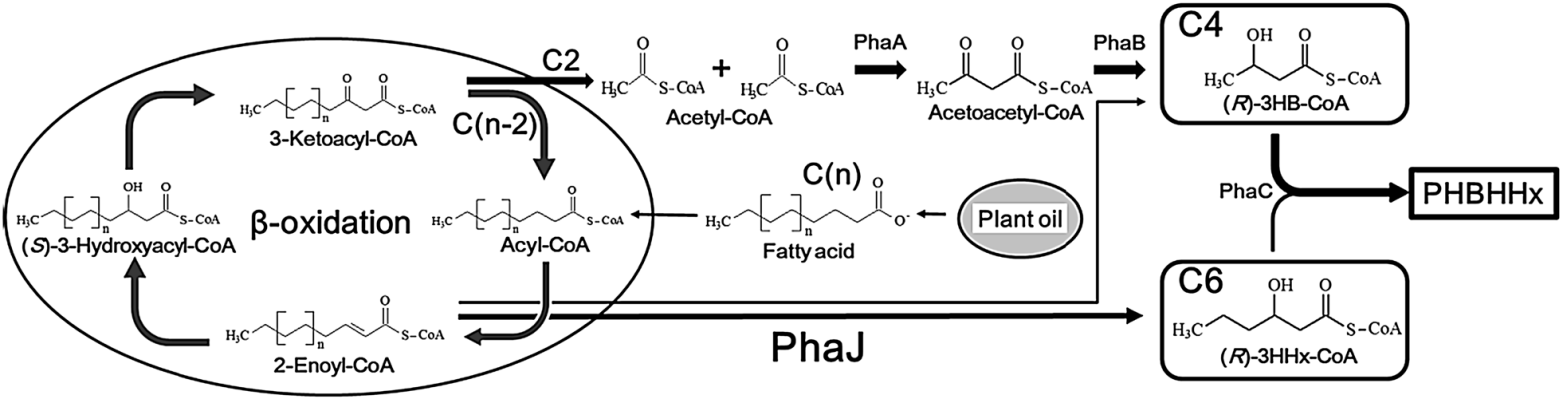
PHBHHx synthesis pathway with *R*-specific enoyl-CoA hydratase. PhaA, 3-ketoacyl-CoA thiolase; PhaB, NADPH-dependent acetoacetyl-CoA reductase; PhaC, PHA synthase; PhaJ, *R*-specific enoyl-CoA hydratase; (*R*)-3HB-CoA, (*R*)-3-hydroxybutyryl-CoA; (*R*)-3HHx-CoA, (*R*)-3-hydroxyhexanoyl-CoA

Previously, the characteristics and role of *R*-specific enoyl-CoA hydratases (PhaJs) in the synthesis pathway of PHA containing medium-chain length monomer units have been reported [20–22]. The PHBHHx synthesis pathway from plant oil via PhaJ is shown in Fig. 5. *Cupriavidus necator* H16 has two *phaJ* genes, which encode enzymes capable of efficiently catalyzing the conversion from β-oxidation intermediate (2-hexenoyl-CoA) to a precursor of the 3HHx monomer unit [(*R*)-3-hydroxyhexanoyl-CoA], named *phaJ4a* and *phaJ4b*, respectively [23]. Thus, we hypothesize that it would be possible to control the 3HHx composition of PHBHHx via the regulation of PhaJ expression when plant oil is used as a carbon source. Therefore, we constructed chromosomal mutant strains, in which the gene expression cassettes described above were placed immediately up-stream of the *phaJ4a* or *phaJ4b* genes in 005dZ126, and then attempted to produce PHBHHx with fine modulated 3HHx composition from palm kernel oil in flask experiments (Table 2).

**Table 2:** PHBHHx production by *phaJ* gene expression-controlled strains

Strain	Dry cell weight (mg/mL)	PHA content (% dry cell weight)	PHA (mg/mL)	Real cell mass^a^ (mg/mL)	3HHx composition (mol%)
005dZ126	19.1 ± 0.1	84.2 ± 0.2	16.1 ± 0.1	3.0 ± 0.1	2.8 ± 0.1
CnC1A	19.3 ± 0.2	84.7 ± 2.0	16.4 ± 0.2	2.9 ± 0.4	5.3 ± 0.1
CnTRPDA	19.1 ± 0.6	84.2 ± 2.7	16.1 ± 0.0	3.0 ± 0.6	7.8 ± 0.0
CnTRPA	19.1 ± 0.3	82.3 ± 1.5	15.8 ± 0.0	3.4 ± 0.3	8.6 ± 0.1
CnUV5DA	19.2 ± 0.5	83.4 ± 0.9	16.0 ± 0.3	3.2 ± 0.3	9.1 ± 0.0
CnUV5A	18.7 ± 0.8	84.0 ± 1.1	15.7 ± 0.4	3.0 ± 0.3	9.9 ± 0.2
CnTRCDA	19.1 ± 0.5	82.1 ± 0.2	15.7 ± 0.3	3.4 ± 0.1	9.6 ± 0.0
CnC1DB	18.8 ± 0.2	85.0 ± 0.6	16.0 ± 0.1	2.8 ± 0.2	5.8 ± 0.2
CnC1B	19.3 ± 0.5	84.2 ± 0.7	16.3 ± 0.3	3.0 ± 0.2	6.7 ± 0.6
CnTRPB	18.8 ± 0.1	84.7 ± 1.3	15.9 ± 0.3	2.9 ± 0.2	10.0 ± 0.1
CnUV5B	19.0 ± 0.0	84.1 ± 1.9	16.0 ± 0.3	3.0 ± 0.4	10.6 ± 0.0
CnTRCB	18.2 ± 0.4	84.0 ± 3.3	15.3 ± 1.0	2.9 ± 0.5	10.7 ± 0.1

The cells were cultured in mineral salt medium with 0.129 w/v% (NH_4_)_2_SO_4_ and 1.5 w/v% palm kernel oil for 72 h. Data represent mean ± SD from three experiments performed in triplicate

^a^Real cell mass = dry cell minus polyhydroxyalkanoates (PHAs)

Within the same gene expression cassette, PhaJ4b brought about a higher 3HHx composition than PhaJ4a. This would result from the differences not only in their specific activities but also in the total expression level of PhaJs in the cells since *phaJ4a* is expressed also under its native promoter, but *phaJ4b* does not [23, 24].

The increased 3HHx compositions of PHBHHx in these recombinant strains, except for CnC1DB, were correlated with the activity determined by a LacZ assay of the expression cassettes inserted upstream of *phaJ4a* and *phaJ4b*. Although CnC1DB produced PHBHHx with a lower 3HHx composition than CnC1B, this was inconsistent with the results from the LacZ reporter assay, but was in agreement with that of the PhaC1 assay (Fig. 2). Therefore, future studies are required to resolve this discrepancy.

**Fig. 6:**
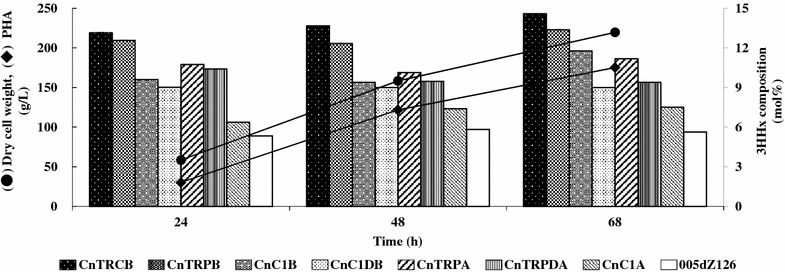
PHBHHx production by high-cell-density fermentation. Dry cell weight (*filled circle*) and PHA (*filled diamond*) in 005dZ126 are shown as representatives, and the values in all the other strains were within ±6 g/L of each of them. The *bars* indicate 3HHx composition of PHBHHx for each strain

In addition, the three PhaJ4a-regulated strains (CnTRPA, CnTRPDA, and CnC1A), four PhaJ4b-regulated strains (CnTRCB, CnTRPB, CnC1B and CnC1DB), and the 005dZ126 strain were used for PHBHHx production by high cell density fermentation. These strains were cultured with feeding palm kernel oil in a jar fermenter for 68 h. The dry cell weight and PHA production under this experimental condition were almost the same as in any strain, and reached approximately 215 and 175 g/L, respectively (Fig. 6). Also in such a high-level production, the rank order of 3HHx compositions of PHBHHx at every sampling point both in the PhaJ4a- and in the PhaJ4b-regulated strains was the same as in the flask experiments, although their values were higher.

In conclusion, we successfully produced, in high yield under the same culture conditions, PHBHHx with various compositions of 3HHx via regulation of PhaJs expression using various expression cassettes.

### Thermal properties of PHBHHx with various 3HHx compositions

Differential scanning calorimetry was used to analyze various PHBHHx samples, each containing different 3HHx compositions produced by the PhaJ expression-controlled strains, as well as the PHBHHx produced by 005dZ126 with PHB as a standard of comparison (Table 3). Melting temperature in the first heating process (*T*_m_) decreased as the 3HHx composition increased. In addition, the crystallinity (*X*_c_) calculated from the melting enthalpy (Δ*H*_m_) of the PHBHHx was much smaller than that of PHB and gradually decreased as 3HHx composition increased. These results indicate that the crystallization of PHB was inhibited by incorporation of 3HHx co-monomer.

**Table 3:** DSC analysis of PHBHHx with various 3HHx composition

3HHx composition (mol%)	First heating process			Cooling process			Second heating process	
	*T*_m_ (°C)	Δ*H*_m_ (J/g)	*X*c (%)	*T*_g_ (°C)	*T*_c_ (°C)	Δ*H*_c_ (J/g)	*T*_cc_ (°C)	Δ*H*_cc_ (J/g)
0 (PHB)^a^	175.1	103.0	70.5	0.4	77.4	80.2	–	–
2.7	150.8	72.9	49.9	−2.3	50.2	25.0	50.3	21.7
5.2	142.0	70.8	48.5	−2.9	41.0	3.6	58.8	47.1
5.9	138.9	69.5	47.6	−2.4	–	–	68.2	43.3
7.1	133.5	66.3	45.4	−2.1	–	–	74.5	27.2
7.9	131.1	67.5	46.2	−2.2	–	–	76.7	20.2
9.1	116.4	65.9	45.1	−3.4	–	–	77.0	12.2
10.0	114.7	60.4	41.4	−3.6	–	–	78.9	1.9
10.8	113.5	55.8	38.2	−4.7	–	–	79.9	1.2

*T*_m_, melting temperature; Δ*H*_m_, melting enthalpy; *X*c, crystallinity; *T*_g_, glass transition temperature; *T*_c_, crystallization temperature; Δ*H*_c_, crystallization enthalpy; *T*_cc_, cold-crystallization temperature; Δ*H*_cc_, cold-crystallization enthalpy

^a^Purchased from Sigma-Aldrich

Furthermore, the crystallization temperature (*T*_c_) and enthalpy (Δ*H*_c_) in the cooling process, as well as the cold-crystallization temperature (*T*_cc_) and enthalpy (Δ*H*_cc_) in second heating process, were also investigated since these parameters reflect the crystallization rate. A large *T*_c_ peak in PHB was observed at 77.4 °C with a Δ*H*_c_ value of 80.2 J/g, which was 78% of Δ*H*_m_, whereas a *T*_cc_ peak was not observed. This suggests that almost all the possible crystallization of PHB under our analysis conditions occurred in the cooling process; that is, PHB was able to crystallize well. On the other hand, no *T*_c_ peak was observed for PHBHHx with ≥5.9 mol% 3HHx, and even Δ*H*_cc_ decreased as the 3HHx composition increased. Therefore, these results suggest that PHBHHx with a high 3HHx composition hardly crystallized under our experimental conditions after once it was melted.

## Discussion

In this study, we attempted to establish a fine gene expression regulation system using combinations of several promoters with either a potential RBS or a disrupted RBS in *C. necator*. The 5′ upstream region of the PHB synthase gene (*phaC1*) has been previously analyzed [19]. The *C. necator* gene *phaC1* is organized in an operon together with the other PHB-biosynthetic genes *phaA* (3-ketoacyl-CoA thiolase) and *phaB* (NADPH-dependent acetoacetyl-CoA reductase), with a relatively long 5′ untranslated region (5′ UTR) of 307 bases in the resulting transcript. The location of the promoter, transcription start site, and the translational initiation codon of *phaC1* have been confirmed experimentally, but not the function of the potential RBS (AGAGAGA located 11 bases upstream of the initiation codon). Therefore, we tested the effect of this potential RBS in regulating protein expression levels, and tried to use it for the construction of gene expression cassettes.

The results of the disruption experiments of the potential RBS by measuring PHA synthase (PhaC1) activity suggest that this RBS sequence has comparatively little ability to interact with the ribosome because the disruption caused insignificant decreases in the resulting activity (Fig. 2). A gene may have multiple RBSs contributing to its expression; for instance, the *pyrC* gene in *E. coli* K-12 has two potential RBS sequences [25]. The attenuation effect of one of them on the expression is significant, but the other is not. Thereby, the *phaC1* gene in *C. necator* may also has other RBS which assumes the leading role.

The gene expression cassettes containing various *E. coli* promoters and the potential RBS of *phaC1* (AGAGAGA) were successfully used in *C. necator*, where their expressions were 5 to 25-fold higher than that of the P_phaC1_RBS (Fig. 4). Moreover, the RBS disruptants were constructed to vary the expression activities. The RBS disruption caused a reduction in expression levels (Fig. 4) and resulted in a lower 3HHx composition, correlating with the activities of the gene expression cassettes inserted immediately upstream of *phaJ* (Table 2; Fig. 6).

In the LacZ reporter assay with P_phaC1_RBS, the disruption of RBS did not cause a decrease in the expression level (Fig. 4). The reason why the attenuating effect was not observed in this experiment is unclear, but the PhaC1 expression and PhaJ expression expected from 3HHx composition with P_phaC1_RBS were reduced by the RBS disruption as described above. Therefore, it may be specific to the combination of P_phaC1_RBS and the *lacZ*, and may be due to influence of some sort of interaction between the long 5′ UTR of P_phaC1_RBS and the *lacZ* gene on the translation or the stability of the mRNA based on the overall structure containing the 5′ UTR (including the RBS region) and the ORF sequence.

The present study demonstrated that the disruption of the potential *phaC1* RBS downstream of the promoters resulted in an attenuation of expression levels. Therefore, this can be used to design other expression cassettes in combination with various promoters to be able to further finely regulate expression levels. In the future, we would like to investigate other functional RBSs in *C. necator*, and the further optimization of gene expression may become available also through the use of RBS libraries.

Furthermore, the thermal properties of PHA are important from a practical application perspective. The incorporation of 3HHx co-monomers in PHA results in a lower melting temperature and lower crystallinity (Table 3), resulting in an increase in elongation [5]. A decrease in the melting temperature is favorable for the improvement of the process window because the melting temperature of PHB is too close to its thermal degradation point [26], and its flexibility is essential for broad practical applications of PHA. Therefore, these characteristics of PHBHHx, with melting temperatures and flexibilities regulated by its 3HHx composition, are preferable as plastic material. On the other hand, consideration should also be given to its ability to crystallize. A lower crystallization rate influences the productivity in the melt processing. The priority of required properties in the plastic material varies by application and processing method. For example, when PHBHHx is used in films, high elongation may be more beneficial than crystallization rate, whereas for blow molding of PHBHHx bottles, a balance of both parameters would be important. Moreover, if other polymers are blended in as a component, the polymers used for blending can compensate for properties that are lacking in the selected PHBHHx. Hence, the control of properties by finely modulating the 3HHx composition is key to using PHBHHx successfully in a wide variety of applications.

Frequent changes in manufacturing conditions should be avoided in the industrial fermentation production for its stable operation. The recombinant *C. necator* strains, constructed in the present study, were able to produce PHBHHx with varying 3HHx composition under the same fermentation conditions in flask (Table 2) and even in high cell density fed-batch culture (Fig. 6). Therefore, we will be able to produce PHBHHx with any required 3HHx composition not only in the laboratory, but also on a commercial scale by using these strains properly, though there is a possibility that the 3HHx composition of PHBHHx will be influenced by the fermenter size, culture conditions such as aeration and agitation, culture medium materials and so on.

## Conclusions

In this work, we showed that the disruption of the potential RBS of the gene *phaC1* results in small decreases in the expression level. Therefore, we utilized this observation to construct the promoter-RBS gene expression cassettes. The expression cassettes comprised of any one of the *E. coli* promoters (P_lacUV5_, P_trc_ and P_trp_) or the *phaC1* promoter in conjunction with the potential RBS or its disruptant exhibited various expression activities in *C. necator*. We then applied these constructs to control the monomer ratio in PHBHHx by regulating PhaJs expression, and demonstrated that they allowed to produce PHBHHx with fine modulated 3HHx composition in recombinant *C. necator*. The observations from this study could raise the possibility of using *C. necator* as a platform for producing useful materials.

## Methods

### Bacterial strains, plasmids, and culture conditions

The strains and gene expression plasmids used in this study are shown in Table 1. The plasmids for chromosomal recombination are shown in Table S1 (Additional file 1). All *E. coli* strains were grown in Luria–Bertani (LB) medium. *Escherichia coli* strains JM109 and S17-1 were used for plasmid construction and as donors in intergeneric conjugation experiments, respectively. All *C. necator* strains were grown in modified basal mineral (MBM) medium as described previously [17].

For enzyme activity assays, the recombinant *C. necator* strains were cultured in 500-mL flask containing 50 mL of the mineral salts medium [27] with 0.129 w/v% (NH_4_)_2_SO_4_ and 1.5 w/v% fructose or palm kernel oil as a sole carbon source for 72 h. To produce PHBHHx, the recombinant *C. necator* strains were cultured using palm kernel oil as described above.

All *E. coli* and *C. necator* strains were cultured at 37 and 30 °C, respectively. When need, kanamycin was added to LB medium and MBM medium at 50 and 100 mg/L, respectively, to maintain the plasmids.

### Construction of recombinant *C. necator* strains for evaluation of gene expression cassettes via enzyme activity assay

The oligonucleotides used for the constructions of the gene expression plasmids are listed in Table S2 (Additional file 1). The *phaC1* expression plasmids pCUP3-P_phaC1_RBS-phaC1, pCUP3-P_phaC1_dRBS-phaC1 and pCUP3-P_phaC1_d50RBS-phaC1 were constructed as follows. The DNA fragment containing P_phaC1_, the following potential RBS, and the *phaC1* gene was obtained by PCR with PphaC1F/phaC1R primers and *C. necator* H16 genomic DNA as a template. This fragment was digested by *Eco*RI and *Spe*I, and then cloned into the *Mun*I and *Spe*I site of pCUP3 to yield pCUP3-P_phaC1_RBS-phaC1. Next, the DNA fragment containing P_phaC1_ and the RBS disruptant (dRBS) was obtained by PCR with PphaC1F/dRBSPphaC1R primers and *C. necator* H16 genomic DNA as a template. The *phaC1* gene fragment was amplified using dRBSphaC1F/phaC1R primers and *C. necator* H16 genomic DNA as a template. These P_phaC1_dRBS and *phaC1* fragments were joined by fusion PCR with PphaC1F/phaC1R, and then digested by *Eco*RI and *Spe*I. This fragment was then cloned into the *Mun*I and *Spe*I site of pCUP3 to yield pCUP3-P_phaC1_dRBS-phaC1.

The resulting plasmid vectors were transformed into the recombinant *C. necator* strain H16/ds containing a disrupted *phaC1* gene. The transformation was performed by electroporation as described previously [17].

The *lacZ* expression plasmids pCUP3-RBS-lacZ, pCUP3-P_lacUV5_RBS-lacZ, pCUP3-P_trc_RBS-lacZ, and pCUP3-P_trp_RBS-lacZ were constructed as follows. The DNA fragment containing potential RBS of *phaC1* and *lacZ* gene was obtained by PCR with MunRBSlacZF/lacZR primers and *E. coli* HB101 genomic DNA as a template. The fragment was digested by *Mun*I and *Spe*I, and cloned into the same site of pCUP3 to yield pCUP3-RBS-lacZ. Next, the P_lacUV5_ was amplified by PCR with lacF/lacUV5R primers and *E. coli* HB101 genomic DNA as a template. The P_lacUV5_ fragment was digested with *Mun*I, and then ligated into the pCUP3-RBS-lacZ digested with the same enzyme to generate pCUP3-P_lacUV5_RBS-lacZ.

Similarly, the P_trc_ and P_trp_ fragments, which were amplified by PCR using pKK388-1 (Clontech) as a template with trcF/trcR or trcF/trpR primers, were digested with *Mun*I and ligated into *Mun*I-digested pCUP3-RBS-lacZ to generate pCUP3-P_trc_RBS-lacZ and pCUP3-P_trp_RBS-lacZ, respectively.

Subsequently, the plasmid pCUP3-P_phaC1_RBS-lacZ was constructed as follows. The DNA fragment containing P_phaC1_ and the following potential RBS was obtained by PCR with PphaC1F/RBSR primers and *C. necator* H16 genomic DNA as a template. The *lacZ* gene fragment was amplified using RBSlacZF/lacZR and *E. coli* HB101 genomic DNA as a template. These P_phaC1_RBS and *lacZ* fragments were joined by fusion PCR with PphaC1F/lacZR, and then digested by *Eco*RI and *Spe*I. The digested fragment was then cloned into the *Mun*I and *Spe*I site of pCUP3 to yield pCUP3-P_phaC1_RBS-lacZ.

Plasmids pCUP3-dRBS-lacZ, pCUP3-P_lacUV5_dRBS-lacZ, pCUP3-P_trc_dRBS-lacZ, pCUP3-P_trp_dRBS-lacZ, and pCUP3-P_phaC1_dRBS-lacZ were all constructed in the same manner as described above, except in the use of primers, where MundRBSlacZF, dRBSPphaC1R, and dRBSlacZF were used instead of MunRBSlacZF, RBSR, and RBSlacZF, respectively.

These plasmid vectors were transformed via electroporation into the recombinant *C. necator* strain 005dZ126, which habors the PHA synthase gene derived form *A. caviae* FA440.

### Construction of chromosomal mutants for the monomer composition modulated PHBHHx production

The oligonucleotides used for the constructions of plasmids for chromosomal recombination are listed in Additional file 1: Table S2. The mutant strain CnUA was constructed as follows. The fragments of *C. necator* DNA corresponding to the regions immediately upstream of the *phaJ4a* open reading frame (ORF) and potential RBS-fused *phaJ4a* ORF were amplified by PCR with J4aUF/J4aUR or RBSJ4aF/J4aR primers and *C. necator* H16 genomic DNA as a template. Furthermore, the fragment containing P_lacUV5_ and potential RBS was amplified by PCR with the J4aUlacUV5F/RBSR primers and pCUP3-P_lacUV5_RBS-lacZ as a template. These three fragments were combined and amplified with the primers J4aUF/J4aR to produce the amplicon containing the sequence upstream of *phaJ4a*, P_lacUV5_, potential RBS of *phaC1,* and the *phaJ4a* ORF. This amplicon was digested with *Smi*I and cloned into the same site of pNS2X-sacB [17] to create the plasmid pNS2X-sacB + P_lacUV5_RBS-J4a. The chromosomal mutant of *C. necator* was created by homologous recombination as described previously [17]. The plasmid pNS2X-sacB + P_lacUV5_RBS-J4a was introduced into the *C. necator* H16 derivative strain 005dZ126, to generate mutant CnUA by conjugation from the donor strain *E. coli* S17-1. Accuracy of the resulting recombination was confirmed via PCR and DNA sequencing.

The same construction strategy was used when preparing the mutant strains, CnUDA, CnUB, CnTRCDA, CnTRCB, CnTRPA, CnTRPDA, CnTRPB, CnCA, CnCB, and CnCDB. The DNA fragments used for construction of pNS2X-sacB + P_lacUV5_dRBS-J4a were generated using J4aUF/J4aUR, dRBSJ4aF/J4aR, and J4aUlacUV5F/dRBSR primers with *C. necator* H16 genomic DNA and pCUP3-P_lacUV5_dRBS-lacZ as templates. The J4bUF/J4bUR, RBSJ4bF/J4bR, and J4bUlacUV5F/RBSR primers with *C. necator* H16 genomic DNA and pCUP3-P_lacUV5_RBS-lacZ as templates were used to generate pNS2X-sacB + P_lacUV5_RBS-J4b; the J4aUF/J4aUR, dRBSJ4aF/J4aR primers and J4aUtrcF/dRBSR with *C. necator* H16 genomic DNA and pCUP3-P_trc_dRBS-lacZ as templates were used to generate pNS2X-sacB + P_trc_dRBS-J4a; the J4bUF/J4bUR, RBSJ4bF/J4bR, and J4bUtrcF/RBStrcR primers with *C. necator* H16 genomic DNA and pKK388-1 as templates were used to generate pNS2X-sacB + P_trc_RBS-J4b; the J4aUF/J4aUR, RBSJ4aF/J4aR, and J4aUtrcF/RBSR primers with *C. necator* H16 genomic DNA and pCUP3-P_trp_RBS-lacZ as templates were used to generate pNS2X-sacB + P_trp_RBS-J4a; the J4aUF/J4aUR, dRBSJ4aF/J4aR, and J4aUtrcF/dRBSR primers with *C. necator* H16 genomic DNA and pCUP3-P_trp_dRBS-lacZ as templates were used to generate pNS2X-sacB + P_trp_dRBS-J4a; the J4bUF/J4bUR, RBSJ4bF/J4bR, and J4bUtrcF/RBSR primers with *C. necator* H16 genomic DNA and pCUP3-P_trp_RBS-lacZ as templates were used to generate pNS2X-sacB + P_trp_RBS-J4b; the J4aUF/J4aUR, RBSJ4aF/J4aR, and J4aUPphaC1F/RBSR primers with *C. necator* H16 genomic DNA and pCUP3-P_phaC1_RBS-lacZ a templates were used to generate pNS2X-sacB + P_phaC1_RBS-J4a; the J4bUF/J4bUR, RBSJ4bF/J4bR, and J4bUPphaC1F/RBSR primers with *C. necator* H16 genomic DNA and pCUP3-P_phaC1_RBS-lacZ as templates were used to generate pNS2X-sacB + P_phaC1_RBS-J4b; the J4bUF/J4bUR, dRBSJ4bF/J4bR, and J4bUPphaC1F/dRBSR primers with *C. necator* H16 genomic DNA and pCUP3-P_phaC1_dRBS-lacZ as templates were used to generate pNS2X-sacB + P_phaC1_dRBS-J4b.

### Enzyme activity assay

β-Galactosidase activity was measured using cell lysates prepared with B-PER Bacterial Protein Extraction Reagent (Pierce Biotechnology, Rockford, IL) and the chromogenic substrate chlorophenol red-β-d-galactopyranoside (CPRG) as previously described [28]. B-PER reagent with protease inhibitors (Halt Protease Inhibitor Cocktail, EDTA-free, Thermo Scientific) was added to the recombinant *C. necator* cell pellets, and after incubation, cell debris was removed by centrifugation. Protein concentrations in the lysates were measured using Takara BCA Protein Assay Kit (Takara Bio Inc., Shiga, Japan). β-Galactosidase assay and the calculation of specific activities were performed according to the manufacturer’s instructions [β-galactosidase assay (CPRG), G-Biosciences, St. Louis, MO].

PHA synthase activities were determined by the 5,5′-dithiobis(2-nitrobenzoic acid) (DTNB) method as previously described [29]. Protein concentrations were measured using the Quick Start Bradford Protein Assay (Bio-Rad Laboratories, Hercules, CA, USA).

### Production of PHBHHx by high-cell-density fed-batch culture

A 5-L jar fermenter (B.E. Marubishi, Co., Ltd.) containing 2 L MB medium [17] was used for high-cell-density fed-batch fermentation. The seed culture was grown over-night at 30 °C in a 500-mL flask containing 100 mL of MBM medium prior to being transferred to the jar fermenter. The jar fermenter operating conditions were as follows: agitation at 500 rpm and aeration rate of 3 L/min, and pH controlled between 6.7 and 6.8 using a 14% aqueous solution of ammonium hydroxide. Cultures were grown at 30 °C for 68 h in a fed-batch process. Palm kernel oil was fed as a carbon source. The feeding rate was 5 g/h from 0 to 24 h, and thereafter, 10 g/h.

### Analysis of dry cell weight and PHA

After culturing, the cells were harvested by centrifugation, washed, and vacuum dried. The dry cell weight was then measured. The cellular content and monomer composition of PHA were determined by gas chromatography (GC) of samples prepared from dry cells as previously described [27].

Differential scanning calorimetry measurements were performed with the EXSTAR 6000 (DSC 6220, SII Nano Technology Inc., Tokyo, Japan). PHA samples were prepared by extraction from dry cells using chloroform and subsequently precipitation with methanol. The analysis was carried out using the thermal conditions as described previously [30]. Samples were first heated at a rate of 10 °C/min to 200 °C and maintained at that temperature for 2 min. Then, they were cooled at a rate of 10 °C/min to −50 °C and maintained at that temperature for 2 min. Subsequently, a second heating was performed at the same rate to 200 °C. The crystallinity was calculated according as

$$X_{\text{c}} = \Delta H_{\text{m}}^{*} /\Delta H_{\text{PHB}}^{0}$$

$\Delta H_{\text{PHB}}^{0}$ is the enthalpy of melting per gram of 100% crystalline 146 J/g [31] and $\Delta H_{\text{m}}^{*}$ is the measured enthalpy of melting for PHB or PHBHHx.

## Additional file

**Additional file 1: Table S1.** Plasmids for chromosomal recombination. **Table S2.** Oligonucleotides used in this study.

## Abbreviations

PHA: polyhydroxyalkanoate
PHB: poly[(*R*)-3-hydroxybutyrate]
PHBHHx: poly[(*R*)-3-hydroxybutyrate-*co*-3-hydroxyhexanoate]
3HHx: (*R*)-3-hydroxyhexanoate
RBS: ribosome binding site
ORF: open reading frame
DSC: differential scanning calorimetry
