# Supplementary material for: Evaluation of gene expression cassettes and production of poly(3-hydroxybutyrate-co-3-hydroxyhexanoate) with a fine modulated monomer composition by using it in Cupriavidus necator
Source: Microb Cell Fact. 2016 Oct 28;15:184. doi: 10.1186/s12934-016-0583-7 (PMC5084369; doi:10.1186/s12934-016-0583-7)
Supplement: Supplementary file 1 — Additional file 1: Table S1. Plasmids for chromosomal recombination. Table S2. Oligonucleotides used in this study. [file 12934_2016_583_MOESM1_ESM.doc]

**Table S1**

Plasmids for chromosomal recombination.

| Plasmid | Description^a^ | Source or  reference |
| --- | --- | --- |
| pNS2X-sacB | Suicide vector, Km^r^ | 17 |
| pNS2X-sacB+P_lacUV5_RBS-J4a | pNS2X-sacB derivative; homologous recombination vector for inserting P_lacUV5_RBS cassette into immediately upstream of *phaJ4a* | This study |
| pNS2X-sacB+P_lacUV5_dRBS-J4a | pNS2X-sacB derivative; homologous recombination vector for inserting P_lacUV5_dRBS cassette into immediately upstream of *phaJ4a* | This study |
| pNS2X-sacB+P_lacUV5_RBS-J4b | pNS2X-sacB derivative; homologous recombination vector for inserting P_lacUV5_RBS cassette into immediately upstream of *phaJ4b* | This study |
| pNS2X-sacB+P_trc_dRBS-J4a | pNS2X-sacB derivative; homologous recombination vector for inserting P_trc_dRBS cassette into immediately upstream of *phaJ4a* | This study |
| pNS2X-sacB+P_trc_RBS-J4b | pNS2X-sacB derivative; homologous recombination vector for inserting P_trc_RBS cassette into immediately upstream of *phaJ4b* | This study |
| pNS2X-sacB+P_trp_RBS-J4a | pNS2X-sacB derivative; homologous recombination vector for inserting P_trp_RBS cassette into immediately upstream of *phaJ4a* | This study |
| pNS2X-sacB+P_trp_dRBS-J4a | pNS2X-sacB derivative; homologous recombination vector for inserting P_trp_dRBS cassette into immediately upstream of *phaJ4a* | This study |
| pNS2X-sacB+P_trp_RBS-J4b | pNS2X-sacB derivative; homologous recombination vector for inserting P_trp_RBS cassette into immediately upstream of *phaJ4b* | This study |
| pNS2X-sacB+P_phaC1_RBS-J4a | pNS2X-sacB derivative; homologous recombination vector for inserting P_phaC1_RBS cassette into immediately upstream of *phaJ4a* | This study |
| pNS2X-sacB+P_phaC1_RBS-J4b | pNS2X-sacB derivative; homologous recombination vector for inserting P_phaC1_RBS cassette into immediately upstream of *phaJ4b* | This study |
| pNS2X-sacB+P_phaC1_dRBS-J4b | pNS2X-sacB derivative; homologous recombination vector for inserting P_phaC1_dRBS cassette into immediately upstream of *phaJ4b* | This study |

^a^ RBS, potential ribosome binding site of *phaC1*; dRBS, RBS disruptant

**Table S2**

Oligonucleotides used in this study.

| Oligonucleotide | Sequence (5´-3´)^a^ |
| --- | --- |
| PphaC1F | GCGCGCGAATTCCCCGGGCAAGTACCTTGCCG |
| phaC1R | GCGCGCACTAGTCGGCTGCCGACTGGTTGAACCAGGCCGGCAGGTCATGCCTTGGCTTTGACGT |
| dRBSPphaC1R | CATGATTTGATTGAGAGAGAGCCGTCACTATTCGAACCGGCTCCG |
| dRBSphaC1F | AATAGTGACGGCTCTCTCTCAATCAAATCATGGCGACCGGCAAAGGCGC |
| MunRBSlacZF | AAGGGCCAATTGCACGTGCAGAGAGACAATCAAATCATGACCATGATTACGGATTCACTGGCCGT |
| lacZR | GCGCGCACTAGTCGGCTGCCGACTGGTTGAACCAGGCCGGCAGGTTATTTTTGACACCAGACCAACTGG |
| lacF | GCGCGCCAATTGGCGCAACGCAATTAATGTGAGTTAGCTC |
| lacUV5R | GCGCGCCAATTGTTTCCTGTGTGAAATTGTTATCCGCTCACAATTCCACACATTATACGAGCCGGAAGCATAAAGTG |
| trcF | GCGCGCCAATTGTGCTTCTGGCGTCAGGCAGCCATC |
| trcR | GCGCGCCAATTGTTTCCTGTGTGAAATTGTTATCCGC |
| trpR | GCGCGCCAATTGTTTCCTGTGTGAAATTGTTATCCGCTGTGAACTTGCGTACTAGTTAACTAGTTCGATGATTAATTGTCAACAGCTC |
| RBSR | CATGATTTGATTGTCTCTCTGC |
| RBSlacZF | AATAGTGACGGCAGAGAGACAATCAAATCATGACCATGATTACGGATTCA |
| MundRBSlacZF | AAGGGCCAATTGCACGTGCTCTCTCTCAATCAAATCATGACCATGATTACGGATTCACTGGCCGT |
| dRBSlacZF | AATAGTGACGGCTCTCTCTCAATCAAATCATGACCATGATTACGGATTCA |
| J4aUF | GCGCGCATTTAAATCGAGGAAGAGATCCTGGCCTTTGC |
| J4aUR | GTCGATAGTCTCCTCTTGACGATAAAGTG |
| RBSJ4aF | GCAGAGAGACAATCAAATCATGCGTACCATCGCATCGCTGG |
| J4aR | GCGCGCATTTAAATTCACCCGTAGCGGCGCGTG |
| J4aUlacUV5F | CACTTTATCGTCAAGAGGAGACTATCGACGCGCAACGCAATTAATGTGAGTTAGC |
| dRBSR | CATGATTTGATTGAGAGAGAGC |
| dRBSJ4aF | GCTCTCTCTCAATCAAATCATGCGTACCATCGCATCGCTGG |
| J4bUF | GCGCGCATTTAAATGCAAGCAGTTCGGCGTGGCG |
| J4bUR | GCTTGCTCTTCCTATTCAGTCAGGG |
| RBSJ4bF | GCAGAGAGACAATCAAATCATGAAGACCTACGAGAACATCGCC |
| J4bR | GCGCGCATTTAAATTCAGGGAAAGCGCCGCAGG |
| J4bUlacUV5F | CCCTGACTGAATAGGAAGAGCAAGCGCGCAACGCAATTAATGTGAGTTAGC |
| J4aUtrcF | CACTTTATCGTCAAGAGGAGACTATCGACTGCTTCTGGCGTCAGGCAGC |
| J4bUtrcF | CCCTGACTGAATAGGAAGAGCAAGCTGCTTCTGGCGTCAGGCAGC |
| RBStrcR | CATGATTTGATTGTCTCTCTGCACGTGCAATTGTTTCCTGTGTGAAATTGTTATCCGC |
| J4aUPphaC1F | CACTTTATCGTCAAGAGGAGACTATCGACCCCGGGCAAGTACCTTGCCG |
| J4bUPphaC1F | CCCTGACTGAATAGGAAGAGCAAGCCCCGGGCAAGTACCTTGCCG |
| dRBSJ4bF | GCTCTCTCTCAATCAAATCATGAAGACCTACGAGAACATCGCC |

^a^ Restriction sites are underlined
